# The Diagnostic Value of Pressure Algometry for Temporomandibular Disorders

**DOI:** 10.1155/2015/575038

**Published:** 2015-03-26

**Authors:** Włodzimierz Więckiewicz, Krzysztof Woźniak, Dagmara Piątkowska, Liliana Szyszka-Sommerfeld, Mariusz Lipski

**Affiliations:** ^1^Department of Prosthetic Dentistry, Faculty of Dentistry, Wroclaw Medical University, 50425 Wroclaw, Poland; ^2^Department of Orthodontics, Pomeranian Medical University of Szczecin, Aleja Powstańców Wielkopolskich 72, 70111 Szczecin, Poland; ^3^Department of Preclinical Conservative Dentistry and Preclinical Endodontics, Pomeranian Medical University of Szczecin, 70111 Szczecin, Poland

## Abstract

The aim of this study is to determine the diagnostic value of pressure algometry in temporomandibular disorders. Two hundred volunteers aged 19.3 to 27.8 (mean 21.50, SD 0.97) participated in this study. An analogue pressure algometer was used for the evaluation of muscle tenderness of the following masticatory muscles: superficial and deep parts of the masseter muscle; anterior and posterior parts of the temporal muscle; and the tissues adjacent to the lateral and dorsal part of the temporomandibular joint capsule. Each patient described the algometry result for the individual components of the masticatory motor system, by selecting each time the intensity of pain on a 100 mm Visual Analogue Scale (VAS) ruler. The area under the receiver operating characteristic (ROC) curve, indicating the discriminatory efficiency for asymptomatic subjects and patients with temporomandibular dysfunction according to the dysfunction Di index, was the largest for the mean pain value (AUC = 0.8572; SEM = 0.0531). The 7.4 VAS cut-off point marked 95.3% specificity for this variable in identifying healthy subjects and 58.4% sensitivity in identifying patients with symptoms of dysfunctions (accuracy 68.1%). Assuming comparable sensitivity (74.9%) and specificity (74.2%) for a diagnostic test, there was test accuracy of 74.5% at the 4.2 VAS cut-off point.

## 1. Introduction

The high universal prevalence of temporomandibular dysfunction among the population means that it is important to look for methods of early diagnosis. In order to limit the negative consequences of adaptive compensation and to prevent prospective decompensation, it is essential to detect pain-free functional disorders in the masticatory system early and to apply therapeutic procedures, which are often simple in nature. Modern instrumental diagnostic methods make it possible to record the dynamics of the symptomatology of functional disorders objectively and quantitatively, as well as enabling the monitoring of therapeutic procedures [1–3].

The diagnosis of temporomandibular dysfunction includes a detailed and focused anamnesis and scrupulous clinical investigation. However, the main symptoms of masticatory motor system dysfunctions such as tenderness, restriction of mandibular movement, or temporomandibular joint noises are evaluated during a routine examination which includes methods characterised by low objectivity [4]. The perception threshold of the examiner is an important constraint in diagnostic efficiency when dealing with discrete signs of dysfunction. Thus, the diagnosis becomes critically dependent on accuracy, that is, the consistency of a practical assessment of a symptom with its true value. However, for many parameters of functional significance, the margin between physiology and pathology is often difficult to observe during a clinical examination. Differentiation between physiology and pathology relies to a greater extent on definition rather than on biometric reality, thus becoming an act of making decisions rather than investigation.

Muscle tenderness on palpation is one of the most important clinical symptoms of masticatory motor system dysfunctions, occurring in about 90% of patients. Manual muscle palpation is the most popular and the most common clinical method for evaluating muscle pain, as well as being at the same time the “gold standard.” However, the main disadvantages of this method include a quantitative assessment of its results and lack of repeatability [5, 6].

The alternative is pressure algometry. This is a diagnostic test which makes it possible to assess muscle pain quantitatively and ensures the repeatability of the diagnostic factor applied.

The aim of this study is to determine the diagnostic value of pressure algometry in temporomandibular disorders.

## 2. Materials and Methods

The research was approved by the Ethics Committee of the Pomeranian Medical University in Szczecin, Poland (number BN-001/45/07), as being consistent with the principles of GCP (Good Clinical Practice). All the patients were informed about the aim and research design and they gave their consent to all of the procedures. After the examination the patients received the information about their condition and function of the masticatory motor system.

Two hundred volunteers (100 females and 100 males), aged 19.3 to 27.8 (mean 21.50, SD 0.97), referred to the Orthodontic Department of the Pomeranian Medical University in Szczecin, participated in this random sampling study. As a result of the application of the adopted exclusion criteria listed in Table 1, 174 of these (93 females and 81 males) qualified for further examinations.

The anamnestic interviews included the patients' general medical history as well as detailed information about their masticatory motor system. They were conducted according to a three-point anamnestic index of temporomandibular dysfunction—Ai (Table 2) [7].

The assessment of the function of the masticatory motor system included clinical examination and pressure algometry. Clinical examination involving visual and auscultatory assessment as well as palpation made it possible to qualitatively and quantitatively evaluate the function of the masticatory system. The clinical index of temporomandibular dysfunction (Di) was used for the analysis of the data obtained from the clinical study (Table 3). The interpretation of the results of the clinical index of temporomandibular dysfunction (Di), based on the total number of points obtained during the tests, was performed according to the following model (Table 4) [7].

An analogue pressure algometer of our own construction was used for the evaluation of muscle tenderness of selected components in the masticatory motor system, such as the superficial and deep parts of the masseter muscle; the anterior and posterior parts of the temporal muscle; and tissues immediately adjacent to the lateral and dorsal parts of the temporomandibular joint capsule. The examination of the abovementioned muscles was performed extraorally, while the dorsal part of the temporomandibular joint was examined by external acoustic pore. Algometer consisted of casing, slide, and steel spring. The housing was constructed of plastic in the shape of a reinforced sleeve finished handle. A precision scale on the cover allow for assessing the force. The slider in the shape of piston had a circular footplate with an overlay of soft rubber. Footplate area size was 1 cm^2^. Force generating element was steel screw spring with specific parameters scaled and described on the scale of the housing. The accuracy of the algometer was 0.5 N. The algometric measurements were performed by the same examiner, alternately on the right and left sides with a constant sequence of examined structures. There was a five-second interval between the examination of the right and left sides. The examination was performed with the patients' dental arches in a slightly open position and the muscles relaxed. During the examination, the footplate of the algometer was always held perpendicular to the skin, in the centre of the belly of the examined muscle, applying a constant force of 18 N (pressure 180 kPa) for duration of 2 s. In the examination of the tissues surrounding the temporomandibular joint, a force of 8 N (pressure 80 kPa) was applied for 2 s. During the test a constant rate of pressure increase was maintained—80 kPa s^−1^. The patients described the algometry results for the individual components of the masticatory motor system, by selecting each time the intensity of pain on a 100 mm Visual Analogue Scale (VAS) ruler.

The Kruskal-Wallis test, the median, and the Mann-Whitney *U* test were used to verify the hypotheses relating to the existence or absence of differences between the mean values of the independent variables (Di group). The correlation between the variables was assessed using Spearman's rank correlation coefficient. The assessment of the accuracy of the classifier (a single variable or the whole model) together with a description of its sensitivity and specificity was based on an analysis of the receiver operating characteristic (ROC) curve. This method made it possible to determine the optimal cut-off points for the specific misclassification costs as well as the* a priori *probabilities for the occurrence of the studied phenomenon. A level of *P* = 0.05 was considered to be statistically significant.

## 3. Results

The findings for the pressure algometry of muscles and temporomandibular joints on the right and left sides for both genders in groups with different severities of temporomandibular dysfunction according to the Di index are presented in Table 5.

**Figure 1:**
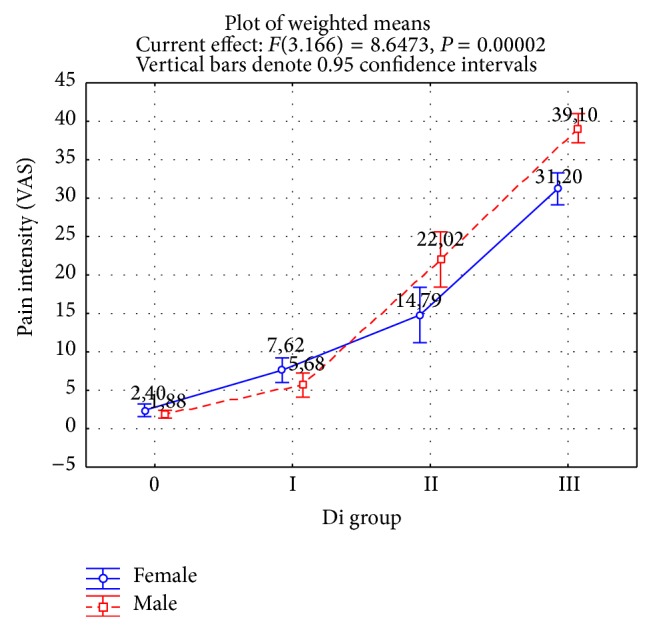
Mean pain value for 12 measurement points depending on the temporomandibular dysfunction index Di.

The analysis of the mean total values of pain defined according to the Visual Analogue Scale (VAS) during the test showed an increase in pain in direct proportion to the severity of temporomandibular dysfunction (*P* < 0.0000; Figure 1). Gender was not a factor affecting the results (*P* < 0.85643). The lowest level of pain was recorded in the group with no dysfunction (Di 0 = 2.13 VAS; *P* < 0.0000). Significantly higher algometry measurements were found in the groups with mild dysfunction (Di 1 = 6.79 VAS; *P* < 0.0000), moderate dysfunction (Di 2 = 18.26 VAS; *P* < 0.0000), and severe dysfunction (Di 3 = 34.85 VAS; *P* < 0.0000).

A regression analysis of the results of algometry and the clinical examination of masticatory motor system dysfunction according to the Di algorithm showed precise correlations between both tests (Table 6). The analysis showed that in algometric tests the mean pain value was a better predictor in terms of functional disorders (*r*_*s*_ = 0.7532; *P* < 0.0000) than the mean absolute difference in pain between the right and left sides (*r*_*s*_ = 0.5529; *P* < 0.0000).

For both analysed variables the degree of correlation increased with respect to muscle and joint pain according to the Di index (*r*_*s*_ = 0.9011 and *r*_*s*_ = 0.7377).

A mathematical analysis of the ROC curve made it possible to compare the sensitivity and specificity of diagnostics tests within the entire range and showed the highest diagnostic efficiency of pressure algometry for the mean pain value (Table 7). The area under the ROC curve, indicating the discriminatory efficiency for asymptomatic subjects and patients with temporomandibular dysfunction according to the Di index, was slightly larger for this variable than in other cases (area under ROC curve (AUC) = 0.8572; standard error of mean (SEM) = 0.0531; *P* < 0.1532). The 7.4 VAS cut-off point marked 95.3% specificity for this variable in identifying healthy subjects and 58.4% sensitivity in identifying subjects with symptoms of dysfunctions (accuracy 68.1%). Assuming a comparable sensitivity (74.9%) and specificity (74.2%) of a diagnostic test, there was test accuracy of 74.5% at the 4.2 VAS cut-off point.

The mean absolute difference in pain between the right and left sides showed a slightly lower diagnostic efficiency in the identification of people with symptoms of dysfunction according to the Di index (AUC = 0.8142; SEM = 0.0495). The 4.1 VAS cut-off point defined 95.5% specificity of this variable in identifying healthy patients and 42.3% sensitivity in identifying subjects with symptoms of dysfunctions (accuracy 68.1%). Assuming comparable sensitivity (68.1%) and specificity (68.4%) for a diagnostic test, there was test accuracy of 68.2% at the 1.7 VAS cut-off point.

## 4. Discussion

Pressure algometry, because of the specific nature of the examination, is dependent on many factors. A crucial element is maintaining constant test conditions. One of the principal local factors which is particularly important in this respect is the invariable position of the algometer in relation to the examined structures. Other important elements include the dynamics of the pressure exerted, the area to which pressure is applied, and the differences between algometers.

A number of short- and long-term clinical experiments conducted by Farella et al. [8] on a group of healthy volunteers and patients with dysfunctions of the masticatory system showed less variation in the repeatability of the pain threshold during pressure algometry than individual variability in this respect. The influence of other factors on the pain threshold did not exceed 25% of the possible variance. Importantly, the prediction interval for the pain threshold relating to various factors is considerably smaller in comparison to the range of differences between healthy subjects and patients with functional disorders of the masticatory system (4–50% of the variance).

High accuracy and precision of pressure algometry was also confirmed in a study by Bernhardt et al. [9]. The study, conducted on a group of 15 healthy volunteers and 15 patients with masticatory motor system dysfunctions, showed high accuracy and repeatability of measurements made using two pressure algometers, with the intraclass correlation coefficient within a range between 0.73 and 0.99.

The results of our own algometric studies showed an increase in the level of pain correlated with the severity of the symtoms of the functional disorders expressed by the Di clinical index of the temporomandibular dysfunction. This was the basis for the evaluation of the diagnostic and discriminant efficiency of this study in relation to the results of a comprehensive clinical trial consistent with the algorithm of the Di index.

The occurrence of the symptom of increased pain on palpation in the structures of the masticatory system in patients with functional disorders has been the subject of numerous studies. Mohn et al. [10] examined the occurrence of pain under experimental conditions in response to transcutaneous electrical stimulation and pressure algometry. Patients with temporomandibular disorders experienced greater pain in response to electrical stimulation and an increase in pain during an isometric contraction, which was not observed in healthy subjects. According to the authors, the increase in pain during an isometric contraction may indicate centralisation of pain sensitivity in patients with temporomandibular dysfunction.

A study by Etöz and Ataoğlu [11] showed a lower pain threshold in a group of 50 people with functional disorders in comparison to a group of 45 healthy people. It also showed a significantly lower pain threshold in patients with a less than 40 mm range of vertical mandibular opening. According to the authors, the lower pain threshold can be a manifestation of subjective symptoms of functional disorders in this group of patients. An algometric study by McBeth and Gratt [12] revealed a significantly greater sensation of pain in the front and middle areas of the temporal muscle, both parts of the masseter muscles, and the lateral surfaces of the temporomandibular joints in a group of 20 patients with functional pain disorders than in a group of 21 people without any symptoms of dysfunction. The respondents assessed the level of pain using a six-point verbal descriptor scale with constant pressure on the muscles (1.8 kg/cm^2^) and temporomandibular joints (0.8 kg/cm^2^). The possibility of discriminating subjects with functional disorders of the masticatory motor system on the basis of algometry was confirmed by Bernhardt et al. [9]. Tests conducted by means of two pressure algometers made it possible to identify the patients with functional disorders and the healthy volunteers.

The possibility of using pressure algometry in the process of diagnosing masticatory system dysfunctions was also confirmed by Visscher et al. [13]. The authors conducted research on a group of 250 respondents, of which 148 manifested subjective pain symptoms, and demonstrated the usefulness of pressure algometry. Their clinical study was based on the principles of a blind sample and involved evaluating through palpation the masseter and temporal muscles as well as the temporomandibular joints. Regression analysis showed that the diagnostic effectiveness of algometry was similar to that of palpation (*r* ^2^ = 0.22 and *r* ^2^ = 0.21, resp.). The highest sensitivity to pain was observed in the masseter muscles and the temporomandibular joints, and the lowest in the temporal muscles.

Variations in the pain threshold for pressure algometry in patients with objective and subjective symptoms of functional disorders (*n* = 50) compared to those without symptoms (*n* = 49) were the subject of research conducted by Silva et al. [14]. An algometric test of the masseter muscles as well as the anterior, middle, and posterior fibres of the temporal muscle showed a significantly lower pain threshold for all the tested muscles in the group with symptoms of functional disorders (*P* < 0.001). In addition, there were significant differences in the level of pain experienced in individual muscles. The lowest threshold of pain was recorded for the masseter muscles, and then for the anterior, middle, and posterior temporal muscle fibres in that order. High (98%) specificity of the algometric test was obtained for the following cut-off points of the muscles: masseter muscles 1.5 kgf/cm^2^; temporal muscles: anterior 2.47 kgf/cm^2^, middle 2.75 kgf/cm^2^, and posterior 2.77 kgf/cm^2^. A ROC curve analysis showed the largest area under the curve (AUC), respectively, for the anterior part (0.92), the middle part (0.90), and the posterior part (0.90) of the temporal muscles and the masseter muscles (0.84). The findings presented by the authors of the study confirmed that the temporal muscles and the masseter muscles have different cut-off points for identifying patients with functional disorders of the masticatory system among a group of healthy patients. Moreover, the greatest diagnostic efficiency of algometric tests was noted for the anterior part of the temporal muscle due to its highest sensitivity amounting to 77%.

The diagnostic value of pressure algometry was the subject of a series of studies conducted by Farella et al. [15]. The studies were conducted by a single physician and were based on the blind sample principle. The algometer used had a pressure area of 1 cm^2^, and the pressure was increased at the rate of 20 kPa/s. The results of this study in a group of 40 women with subjective symptoms of functional disorders of the masticatory system showed a significantly lower pain threshold for the masseter and temporal muscles than in a group of 40 healthy women (*P* < 0.001). The pain threshold for the masseter muscles and the anterior parts of the temporal muscles in women with functional disorders was about 40–50% lower than in the control group. In addition, muscle pain on palpation was significantly greater on the side with more intense subjective symptoms. The placement of the cut-off point one standard deviation below the mean value of the pain threshold in the control group determined the sensitivity and specificity of pressure algometry for the masseter muscles at, respectively, 67% and 85%, and for the temporal muscles at 77% and 87%. The authors also determined the probability of the occurrence of the condition in patients who had a positive result in the algometric test, that is, the positive predictive value of the test, which is closely correlated with the prevalence of a specific disease in the population. From adopted prevalence of masticatory system dysfunctions at a level of 17.4%, the positive predictive value for the temporal muscles was 55% and for the masseter muscles 48%. Thus, the use of pressure algometry for screening may generate 50% false-positive results in a population of women. Assuming, however, the prevalence of functional disorders at a level of 33%, the positive predictive value for temporal muscles would be 74%, and for the masseter muscles 68%. Nevertheless, there is still the possibility of approximately 30% false-positive results, which according to the authors significantly reduces the possibility of using algometry for screening.

In conclusion, it is important to emphasise the possibility of analysing algometric tests in terms of both the intensity and the symmetry of pain within homonymous structures. Mathematical analysis also confirmed significant correlations between the results of algometric tests and a clinical trial consistent with the algorithm of the Di index. Furthermore, the intensity of pain indicated on a Visual Analogue Scale and its asymmetry may be a predictor of functional changes in the masticatory organ. A crucial element is also the stability and repeatability of the applied pressure, which enhances the credibility of the results obtained.

## 5. Conclusions

Respecting the limitations of this study, the evaluation of pressure algometry demonstrated its diagnostic effectiveness with regard to symptoms of temporomandibular dysfunction.

## Figures and Tables

**Table 1:** The exclusion criteria adopted in anamnesis.

Exclusion criteria	Number of the excluded subjects
Depressive disorders	0
Pain in other parts of the body	4
Inflammations	3
Taking painkillers and antidepressants	1
Periodontal diseases	1
Completed treatment of masticatory motor system dysfunctions	2
Completed orthodontic treatment	15

Total	26

**Table 2:** Anamnestic index of temporomandibular dysfunction (Ai).

Ai	Symptoms
I	No subjective symptoms of temporomandibular dysfunction—no symptoms reported by patient.

II	Mild symptoms of temporomandibular dysfunction—temporomandibular joint noise, feeling of “jaw fatigue” (fatigue of masticatory muscles), and feeling of “jaw rigidity” (increased tone of masticatory muscles).

III	Severe symptoms of temporomandibular dysfunction—restricted mouth opening, painful lower jaw movements, temporomandibular joint pain, masticatory muscle pain, temporomandibular joint luxation, and lockjaw.

**Table 3:** Clinical index of temporomandibular dysfunction (Di).

Di	Symptoms
Mandibular movements	
0	Normal range
1	Small reduction in amplitude
5	Large reduction in amplitude

Temporomandibular joint function	
0	Smooth, noiseless abduction and adduction of mandible, trajectory asymmetry <2 mm
1	Noise in one joint or both joints during abduction and adduction of mandible, trajectory asymmetry >2 mm
5	Abduction of mandible impossible and/or luxation

Masticatory muscle pain	
0	No tenderness
1	Tenderness of 1–3 sites
5	Tenderness of 4 and more sites

Temporomandibular joint pain	
0	No tenderness
1	Unilateral or bilateral tenderness
5	Unilateral or bilateral tenderness of the dorsal surface of joint

Pain during movement of mandible	
0	No pain
1	Pain during one out of all possible movement directions
5	Pain during more than one out of all possible movement directions

**Table 4:** Interpretation of the clinical index of temporomandibular dysfunction (Di).

Range	Severity of dysfunction	Description
0	Di 0	No dysfunction
1–4	Di I	Mild dysfunction
5–9	Di II	Moderate dysfunction
10–25	Di III	Severe dysfunction

**Table 5:** Muscle and temporomandibular joint algometry [VAS] findings depending on the temporomandibular dysfunction index Di.

Side/gender	Di group											
	0			I			II			III		
	*n*	Mean	SD	*n*	Mean	SD	*n*	Mean	SD	*n*	Mean	SD
Superficial part of masseter muscle												
Left												
Females	22	5.57	5.71	39	14.47	10.10	25	23.94	18.08	7	41.29	22.13
Males	23	4.76	4.07	29	10.31	9.06	23	41.50	21.81	6	40.33	5.43
Total	**45**	**5.16**	**4.90**	**68**	**12.70**	**9.82**	**48**	**32.35**	**21.64**	**13**	**40.85**	**16.04**
Right												
Females	22	6.52	6.72	39	19.32	14.78	25	34.88	28.69	7	61.00	13.56
Males	23	5.15	3.41	29	11.53	8.57	23	48.43	24.80	6	60.67	5.57
Total	**45**	**5.82**	**5.28**	**68**	**16.00**	**13.02**	**48**	**41.38**	**27.48**	**13**	**60.85**	**10.25**

Deep part of masseter muscle												
Left												
Females	22	2.73	3.98	39	7.90	8.81	25	24.28	16.75	7	35.14	22.52
Males	23	3.54	4.09	29	9.14	10.54	23	39.35	19.89	6	23.00	7.97
Total	**45**	**3.14**	**4.01**	**68**	**8.43**	**9.53**	**48**	**31.50**	**19.66**	**13**	**29.54**	**17.88**
Right												
Females	22	3.39	3.53	39	7.41	8.49	25	19.98	12.03	7	29.57	15.40
Males	23	1.59	1.10	29	9.17	10.26	23	31.17	15.67	6	47.33	4.08
Total	**45**	**2.47**	**2.72**	**68**	**8.16**	**9.25**	**48**	**25.34**	**14.86**	**13**	**37.77**	**14.51**

Anterior part of temporal muscle												
Left												
Females	22	0.84	0.54	39	4.13	4.97	25	7.70	7.24	7	5.29	1.60
Males	23	0.76	0.77	29	2.60	2.58	23	13.48	10.83	6	9.00	10.10
Total	**45**	**0.80**	**0.66**	**68**	**3.48**	**4.17**	**48**	**10.47**	**9.50**	**13**	**7.00**	**6.89**
Right												
Females	22	1.07	0.94	39	4.87	5.39	25	9.78	10.04	7	19.43	10.06
Males	23	1.07	1.31	29	3.90	4.31	23	17.26	11.97	6	39.00	7.35
Total	**45**	**1.07**	**1.13**	**68**	**4.46**	**4.95**	**48**	**13.36**	**11.52**	**13**	**28.46**	**13.28**

Posterior part of temporal muscle												
Left												
Females	22	1.48	1.26	39	5.37	5.93	25	6.50	5.24	7	10.29	8.22
Males	23	0.61	0.81	29	3.17	3.94	23	7.02	5.09	6	22.00	1.55
Total	**45**	**1.03**	**1.13**	**68**	**4.43**	**5.25**	**48**	**6.75**	**5.12**	**13**	**15.69**	**8.47**
Right												
Females	22	1.43	1.17	39	4.80	5.18	25	9.82	7.52	7	18.86	18.87
Males	23	0.98	0.71	29	3.67	4.41	23	9.39	6.37	6	14.00	9.36
Total	**45**	**1.20**	**0.98**	**68**	**4.32**	**4.86**	**48**	**9.61**	**6.93**	**13**	**16.62**	**14.86**

Lateral surface of temporomandibular joint												
Left												
Females	22	0.89	1.34	39	4.65	8.39	25	6.24	7.58	7	10.00	8.60
Males	23	1.43	1.37	29	2.71	2.65	23	11.87	8.34	6	32.17	2.71
Total	**45**	**1.17**	**1.37**	**68**	**3.82**	**6.62**	**48**	**8.94**	**8.37**	**13**	**20.23**	**13.13**
Right												
Females	22	1.61	1.75	39	5.49	6.49	25	10.00	10.31	7	29.29	14.75
Males	23	0.93	1.15	29	3.93	3.39	23	17.11	13.04	6	48.00	13.55
Total	**45**	**1.27**	**1.50**	**68**	**4.83**	**5.41**	**48**	**13.41**	**12.12**	**13**	**37.92**	**16.72**

Dorsal surface of temporomandibular joint												
Left												
Females	22	1.82	2.37	39	6.98	8.70	25	12.80	11.04	7	57.57	26.65
Males	23	0.54	0.42	29	3.53	2.82	23	13.87	10.65	6	62.33	17.91
Total	**45**	**1.17**	**1.79**	**68**	**5.51**	**7.01**	**48**	**13.31**	**10.76**	**13**	**59.77**	**22.25**
Right												
Females	22	1.45	0.87	39	5.99	6.17	25	11.54	8.23	7	56.71	23.68
Males	23	1.17	0.90	29	4.50	4.31	23	13.83	8.80	6	71.33	15.91
Total	**45**	**1.31**	**0.89**	**68**	**5.35**	**5.47**	**48**	**12.64**	**8.50**	**13**	**63.46**	**21.05**

**Table 6:** Correlations between algometry findings and temporomandibular dysfunction index Di.

Algometry	Total Di value		Muscle and joint pain in Di	
	*r*_*s*_	*P* value	*r*_*s*_	*P* value
Mean pain value^1^	0.7532	0.0000	0.9011	0.0000
Mean absolute difference in pain between right and left side^1^	0.5529	0.0000	0.7377	0.0000

^1^Mean value for 12 measurement points.

**Table 7:** Data for some cut-off points in algometry as discriminators for patients without symptoms of temporomandibular dysfunction or with Di group I, II, or III.

Variable	Parameter	Sensitivity = specificity	Specificity = 95%	AUC (SEM)
Mean pain value^1^	Cut-off point	4.2 VAS	7.4 VAS	0.8572 (0.0531)
	Sensitivity	74.9%	58.4%	
	Specificity	74.2%	95.3%	
	Accuracy	74.5%	68.1%	

Mean absolute difference in pain between right and left side^1^	Cut-off point	1.7 VAS	4.1 VAS	0.8142 (0.0495)
	Sensitivity	68.1%	42.3%	
	Specificity	68.4%	95.5%	
	Accuracy	68.2%	52.5%	

^1^Mean value for 12 measurement points.

AUC: area under ROC curve, SEM: standard error of mean.
